# Assessing the Best Gap-Filling Technique for River Stage Data Suitable for Low Capacity Processors and Real-Time Application Using IoT

**DOI:** 10.3390/s20216354

**Published:** 2020-11-07

**Authors:** Antonio Madueño Luna, Miriam López Lineros, Javier Estévez Gualda, Juan Vicente Giráldez Cervera, José Miguel Madueño Luna

**Affiliations:** 1 Aerospace Engineering and Fluid Mechanical Department, University of Seville, 41013 Seville, Spain; 2 Design Engineering Department, University of Seville, 41013 Seville, Spain; mlopezlineros@us.es; 3 Engineering Projects Area, Department of Rural Engineering, University of Córdoba, 14071 Córdoba, Spain; jestevez@uco.es; 4 Agronomy Department, University of Córdoba, 14071 Córdoba, Spain; ag1gicej@uco.es; 5 Agronomy Department, Institute for Sustainable Agriculture (IAS)—Spanish National Research Council (CSIC), Alameda del Obispo, 14080 Córdoba, Spain; 6 Graphics Engineering Department, University of Seville, 41013 Seville, Spain; jmadueno@us.es

**Keywords:** gap-filling, river stage data, cubic splines, radial basis functions, multilayer perceptron, Arduino, Raspberry pi, IoT

## Abstract

Hydrometeorological data sets are usually incomplete due to different reasons (malfunctioning sensors, collected data storage problems, etc.). Missing data do not only affect the resulting decision-making process, but also the choice of a particular analysis method. Given the increase of extreme events due to climate change, it is necessary to improve the management of water resources. Due to the solution of this problem requires the development of accurate estimations and its application in real time, this work present two contributions. Firstly, different gap-filling techniques have been evaluated in order to select the most adequate one for river stage series: (i) cubic splines (CS), (ii) radial basis function (RBF) and (iii) multilayer perceptron (MLP) suitable for small processors like Arduino or Raspberry Pi. The results obtained confirmed that splines and monolayer perceptrons had the best performances. Secondly, a pre-validating Internet of Things (IoT) device was developed using a dynamic seed non-linear autoregressive neural network (NARNN). This automatic pre-validation in real time was tested satisfactorily, sending the data to the catchment basin process center (CPC) by using remote communication based on 4G technology.

## 1. Introduction

As in the case of hydrologic variables such as precipitation [1], complete historical records are necessary in river stage data sets. In the current climate change context, complete time-series of these data are essential for a comprehensive study of the evolution of the magnitude of changes. An adequate water resources management process is crucial to minimize the impact of extreme events [2]. One of the main problems in the analysis of time series is the absence of data, with gaps of different widths, number of missing data and frequency, which makes the model identification harder and prevents the adoption of common validation procedures, usually applied to complete data sets [3,4,5,6]. These deficiencies in hydrologic time series are usually due to the malfunctioning of monitoring equipment, the occurrence of anomalous natural phenomena and data transmission storage and retrieval process issues [7]. Sometimes, the solution of these problems is not instantaneous, demanding the intervention of qualified personnel at the measuring point or the development of specific methods for the detection of spurious signals in datasets from automatic acquisition systems [5]. Occasionally, when there are no data, they can be recovered by making a backup from the data loggers, although this is not always possible. Nevertheless, even when the back-up is made, quality of data sets could be preserved. To avoid these drawbacks, data recovery systems or infilling procedures are needed.

There are different gap-filling procedures usually specific for the nature of the hydrologic variable under study. River stage data present some stationarity so that interpolation methods can be directly applied in order to rebuild the data set. This can be achieved with statistical methods [8,9], or by applying artificial neural networks (ANNs) [7]. The use of the auto regressive moving average (ARMA) method [10,11], allows the estimation of missing data in stationary sets, but the problem with these easy and available interpolation methods is that they needs a pre-identification of the data-set model [12] which could be an inconvenience for the real time validation. On the other hand, there are other procedures such as the Gray model [13], wherein the assumption in the probability distribution of data is not required and only three data points are required for the modeling; the computation effort required is small for the construction of the model and it is highly adaptive to system dynamics behavior. Other approximations [14] have faced this issue using an appropriate weighting of the estimated values generated by two autoregressive processes operating: in the forward and backward directions of time [15,16].

The main aim has been to design the best gap-filling techniques updated with the new social environment and new electronic products which associated with a good Internet of Things (IoT) technique could provide real-time and cheaper maintenance, being the citizenry and scientific community the first to benefit. This gap-filling technique for river stage data sets, which had to be developed to efficiently run on low-cost architecture (e.g., Arduino and Raspberry Pi), allowing easy positioning at both single and multiple locations. The use of small processors, with low energy requirement, and reduced computing potential, is justified in the case of outdoor sensors, which have to be supplied with batteries of great size to be lodged in a large gauge cabin. With these low-cost, low-consumption devices the gauge cabin could be reduced, and, the most important, the system will have greater autonomy even in critical cases (electrical supply failure). In addition, although the price of processors is getting down, the largest size and price of a personal computer still represents an inconvenient. Some examples are reported in Table 1.

To achieve this, three different customized gap-filling estimation methods with increasing processor speed requirements were taken into account. The first method adopts cubic splines [17,18,19,20,21,22,23]. The second one interpolates with radial basis functions [24,25,26,27,28,29,30]. Finally, the third method is an ad hoc neuronal network: the multilayer perceptron (MLP) [31,32,33,34,35,36,37,38,39,40], expecting in this case that fitting weighed and biased algorithms would be compatible with a simple calculation based on metaheuristic techniques such as simulated annealing (SA) or particle swarm optimization (PSO), [41]. To compare the results of these three different methods, a statistical study was carried out with random samples from a measurement point placed in the Andalusian Guadalquivir river basin, (A08_101). This work assesses the previously cited gap-filling techniques in order to find out the best method depending on the size of the gap in river stage data.

On the other hand, in order to include this pre-validation system, an Internet of Things (IoT) device has been developed, being capable of making a remote 4G connection and returning the data that have already been corrected.

## 2. Materials and Methods

### 2.1. Study Area and Data Source for Data Correction

In this study, river stage data from one-gauge station (A08_Mengíbar), located in the Basin of Guadalquivir River (Southern Spain), were used. This station belongs to the Automatic Hydrologic Data Collection System (SAIH), controlled by the Spanish goverment [42].

These river stage records managed by the Catchment Basin Process Center (CPC), have a 15-min sampling. The data are collected by a Vegaplus 62 radar sensor [43], with a 4–20 mA output loop signal, 35 m measurement range, +/− 2 mm of error accuracy and resolution of 12 bits.

One of the inherent features in these data records is that any minor variation is recorded due to the accuracy of the radar system, in contrast with the data recorded by mechanic sensors based on float level systems, which return smoothed values.

**Figure 1:**
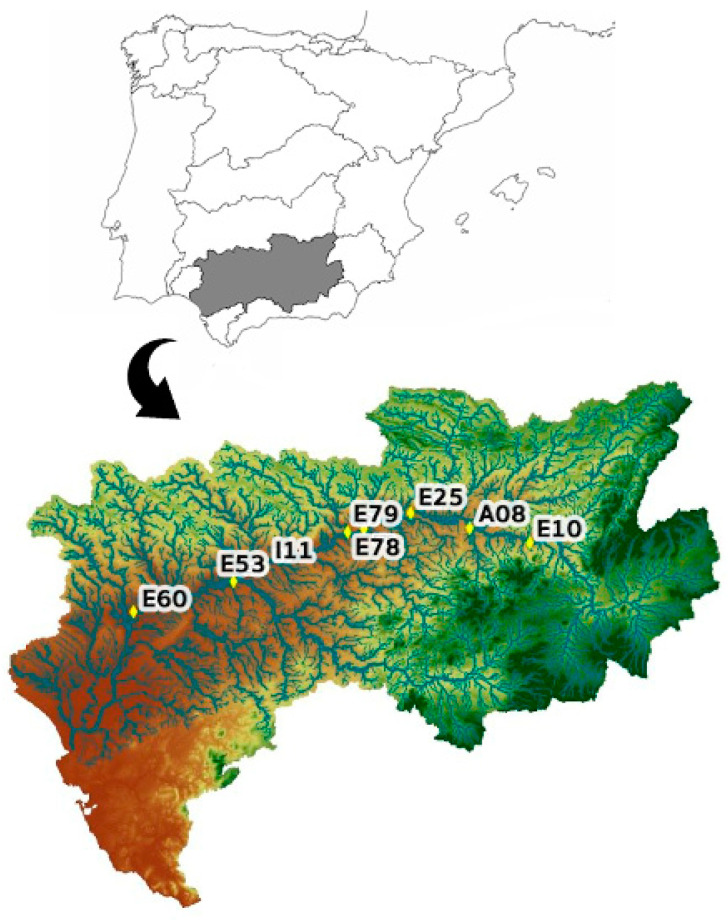
Control points on the main Guadalquivir’s river axis: E10 (Pedro Marín), A08_101 (Mengibar), E25 (Marmolejo), E78 (El Carpio), E79 (Villafranca), I11 (Fuente Palmera), E53 (Peñaflor), and E60 (Alcalá del Río).

This station, A08_101 (Mengíbar), has been selected due to: (i) its location in the main axis of Guadalquivir river at the boundary of the influence of Atlantic winds, Figure 1, (ii) its relevance in being the data source most consulted on the Web, and (iii) its strategic value for flooding control, downstream of a dam.

### 2.2. Control Point Used to Test the Alternative Pre-Validation System Developed

The selected control point to verify the alternative pre-validation system is part of the Guadalquivir Alert Hydrological Information System (SAIH in Spanish) network (A17 Genil-Écija), with UTM coordinates: 37.5580723149, −5.0777982501, zone 30. This selection was made due to (i) the ease of periodic access, so that any incident could be resolved in a timely manner relatively short; (ii) good 4G coverage to have a 24-h remote connection with the equipment; (iii) enough physical space to install all the equipment; (iv) possibility of powering the equipment from the point’s own 24 V batteries; and (v) ability to double the level sensor output with a galvanic separator. After consulting with the SAIH management and taking into account the strategic importance of the point due to the risk of frequent floods, this point was selected from among those suggested by the agency.

**Figure 2:**
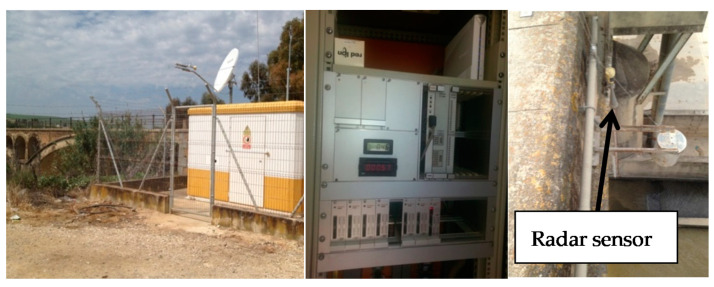
A17 Genil-Écija checkpoint booth with satellite dish for satellite connection with Hispasat 1ª, SAIH equipment (SAINCO/Telvent) and the Vegaplus 62 radar sensor.

In Figure 2, an image of the external booth of the control point is shown, of the SAIH equipment [44], installed inside and finally of the Vegaplus 62 radar sensor in the Iron Bridge (Genil River).

### 2.3. Gap-Filling Techniques

The World Meteorological Organization [45] proposed some general criteria which have been adapted to the implemented measurement system recording and analyzing these data. Consequently, data records are classified in 6 different types characterized by a flag (Table 2).

These flags allow an analysis of the different kind of gaps present in the data set, applying the three techniques detailed below.

In this work, three gap-filling methods have been used with a similar validity to the methods ARIMA or Holt-Winters [46], with the possible advantage of a greater simplicity, which is required for in-situ setups. In addition, it is important to emphasize that the data once formally validated in the central database unit are returned to the remote device (Arduino/RPi). These devices will be continuously feeding with quality data and having, in the worst case, up to 2-week of pre-validated data processed by themselves.

#### 2.3.1. Cubic Splines

Originally, spline was a term used for flexible rulers that were bent to pass through a number of predefined points, “knots”, hence the name S-line. Since several decades, the method has been widely applied in industrial design, especially in automobile manufacturing [47].

Splines are piecewise polynomials, like Lagrange or Hermite polynomials, maintaining continuities at the knots in only primary functions but their derivatives down to a certain order, which makes them very useful as interpolators, and, consequently in Computer Aided Design [48,49,50]. Due to their simplicity, cubic splines are a widely used tool [51].

#### 2.3.2. Radial Basis Functions

Radial basis functions (RBF) are real-valued functions whose argument is the distance from the origin [52]. Let $x_{1}{,\;x}_{2}$,…$\;x_{N}\in \Omega \subset R^{n}$ be a given set of nodes. Given interpolation data values $y_{1}{,y}_{2}$,…$\;y_{N}\in R,$ at data locations $x_{1}{,x}_{2}$,…${\;x}_{n}$, RBF, g_j_(x) are defined as follows:

(1) $$g_{j}\left(x\right)\equiv g\left(\|x-x_{j}\|\right)\in R,\;j\;=\;1,\ldots ,N$$

where $\left\|x-x_{j}\right\|$ is the Euclidean distance. RBFs are used to interpolate scattered data. The RBF interpolant is:

(2) $$F(x)\;=\;\overset{N}{\underset{j\;=\;1}{\sum }}\alpha_{j}{\cdot g}_{j}\left(x\right){+\alpha }_{N+1}$$

It is obtained by solving the system of N+1 linear equation, for N + 1 unknown expansion coefficients, α_j_ an independent term, α_N+1_. Among the huge amount of RBF functions, those most commonly used are:

(3) $$\mathrm{thin-plate}\;\mathrm{splines}:\;g\left(x\right)\;=\;\left\|x-x_{j}\right\|\cdot \ln\left(\|x-x_{j}\|\right)$$

(4) $$\mathrm{linear}\;\mathrm{splines}:\;g(x)\;=\;\left\|x-x_{j}\right\|$$

(5) $$\mathrm{cubic}\;\mathrm{splines}:\;g\left(x\right)\;=\;{\left\|x-x_{j}\right\|}^{3}$$

(6) $$\mathrm{gaussian}\;\mathrm{splines}:\;g\left(x\right)\;=\;\exp\left(-\frac{\left\|x-x_{j}\right\|}{c_{j}^{2}}\right)$$

(7) $$\mathrm{multiquadric}\;\mathrm{splines}:\;g\left(x\right)\;=\;\sqrt{1+\frac{{\left\|x-x_{j}\right\|}^{2}}{c_{j}^{2}}}$$

#### 2.3.3. Multilayer Perceptrons

The use of perceptrons is a reasonable way to reduce the risk of incorporation spurious data [3].

**Figure 3:**
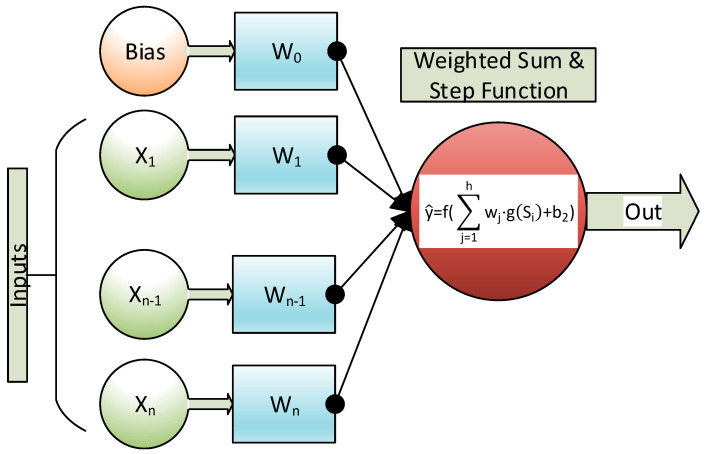
Scheme of a conventional multilayer perceptron.

A conventional multilayer perceptron (MLP) [53] has three layers: an input layer, one or more hidden layers and an output layer. In a traditional MLP the information, or input signal, is moved forward as shown in Figure 3. The MLP output is a node or neuron with a linear activation function (f). On the other hand, hidden layers have a sigmoid activation function (g):

(8) $$\hat{y}\;=\;f(\sum_{j=1}^{h}w_{j}\cdot g(S_{i})+b_{2})$$

This kind of model is usually trained with a back-propagation algorithm (BP). These neural networks are universal approaches of any continuous function, as long as there is at least one hidden layer. There are no rules for the selection of the best number of nodes in the hidden layer in order to achieve a certain level of error [54]. The updating weight and bias values has been calculated according to the Levenberg-Marquardt optimization (LM).

### 2.4. Gap Filling Techniques Used

With the aim of using small processors, with low energy requirement and reduced computing potential, two types of studies have been carried out:

#### 2.4.1. Test Type I (Scattered Gaps)

**Figure 4:**
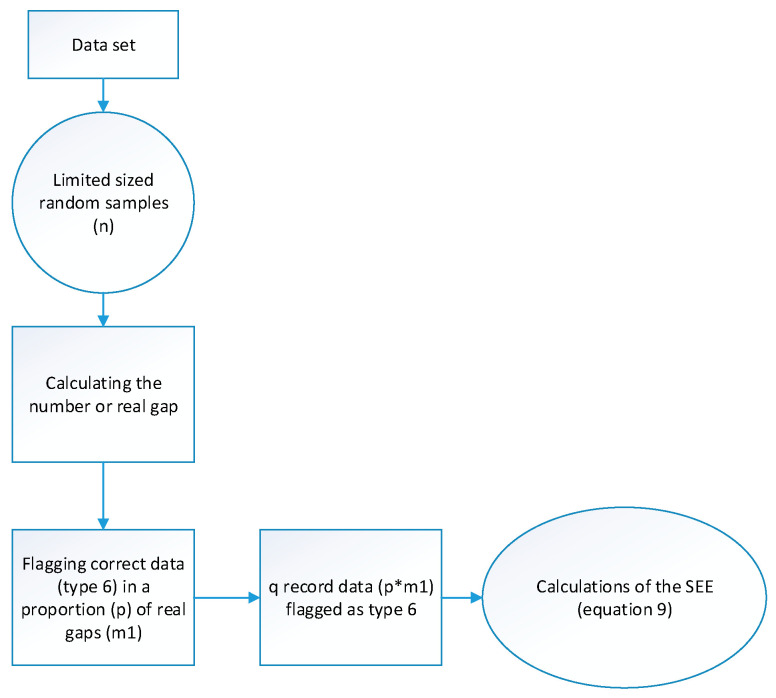
Block diagram followed in Test I.

In order to analyze scattered gaps comparing the differences between the three different methods proposed, size-limited random samples (n) from the data set were chosen as a first test. The maximum number of gap-data in the set should not be more than a *m* fraction of *n, m*1, being the number of gaps found in that random sample no more than m fractions of *n.* In addition, a new type 6 flag was added to fraction, *p*, of *m*1, Figure 4.

Therefore, if we have a random sample *n* = 1000 and a gap fraction *m* = 0.1, the number of maximum gaps should be 100. Thus, if *m*1 = 70 gaps found and the fraction of data to be marked is *p* = 0.80, then q = 70 × 0.8 = 56 Type 6 data will be added. These data are used to estimate the goodness-of-fit of the curve in each one of the different methods. This comparison is evaluated by the standard error of the estimate (SEE), considering the marked data as Type 6 and following [55]:

(9) $$\mathrm{SEE}\;=\;\sqrt{\frac{\sum_{i\;=\;1}^{q}{e\_\mathrm{spline}}_{i}{}^{2}}{q-2}}$$

The e_spline represents the difference between the original, correct value and the value estimated by the method used in each case. In this example, q = 56. Additional information on the software developed for this study can be found in the Appendix 1 [56].

As mentioned before, the gap-filling has been calculated by three methods: cubic splines (pchip), radial basis functions with five variants of these (lineal, Gaussian, quadratic, multiquadric and thin plate spline), using the Chirokov algorithm, [57] and multilayer perceptron.

#### 2.4.2. Test Type II (Multiple Gaps)

**Figure 5:**
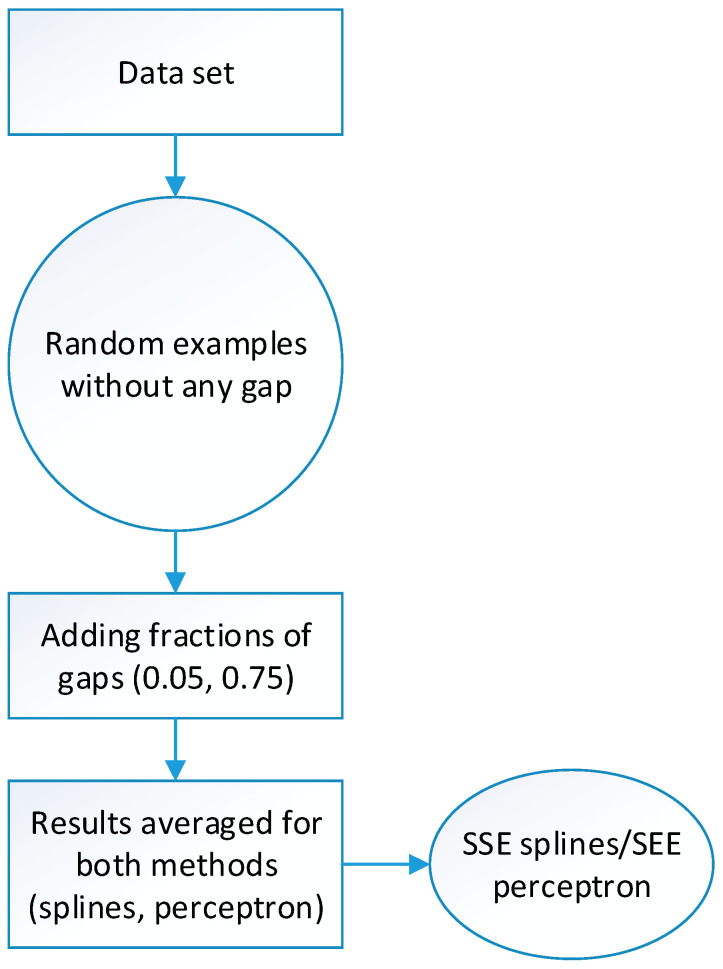
Block diagram followed in Test II.

As will be shown in Section 3, MLP may be suitable for filling multiple gaps. A test has been developed to analyze its performance. The sequence followed in this second test is shown in Figure 5. Additional information on the software developed for this study can be found in the Appendix 2 [56].

### 2.5. Using a NARNN with Dynamic Seed

A non-linear autoregressive neural network with external input (NARNN) [58] has been used with a seed that increases gradually during the validation process [3], whose performance has been contrasted by comparing it with the standard methods applied in validating the data of a float sensor to which errors of known magnitude have been added.

The great disadvantage of this method is that a seed size is reached in the recursive training of the NARNN that demands such computing power that it is not of practical application. For this reason, a new dynamic seed will be used here such that:

(10) $$\mathrm{seed}\in \left[t_{1+k}{,\;t}_{2+k}\right]\;\forall \;k\in \left[0,\;m\right]$$

where m is the number of data to validate, and t_2_−t_1_ is the number of data of the seed.

Thus, if the data that feed the NARNN are correct, it will be able to make correct predictions for the t + 1 data. If the data that arrives is not correct, the NARNN may issue an alert signal so that the operator in charge of analyzing the data warns of the incident and makes the corresponding correction. The NARNN will have the capacity, given its natural tolerance to failures, to support a fraction of erroneous data with which it will provide feedback. Ideally, in the practical validation process, the data marked as erroneous should be extracted from the series from time to time, corrected, so the NARNN always is retrained with correct data in order to achieve optimal use of it.

**Figure 6:**
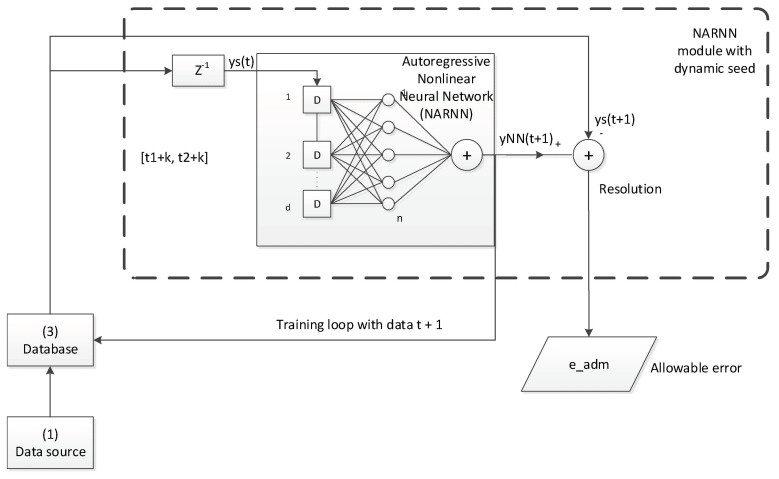
Dynamic seed NARNN validation algorithm.

To analyze the behavior of this dynamic NARNN, it has been trained with validated data from the 21 days that the trial lasted and its resolution has been quantified by varying its parameters: cadence between data (3600 s,..., 60 s), the delay of feedback (1, 6, 11, 16, 21 and 24) and the ratio between the data used for training and the total of those acquired (0.25,…, 0.95), the difference up to 1 (0.75,…, 0.05) is the fraction that would be used for validation. Figure 6 describes the operation of the validation algorithm with a dynamic seed NARNN. Additional information on the software developed for this study can be found in Appendices 3 and 4 [56].

### 2.6. Alternative Electronic Equipment Developed for IoT Communication

**Figure 7:**
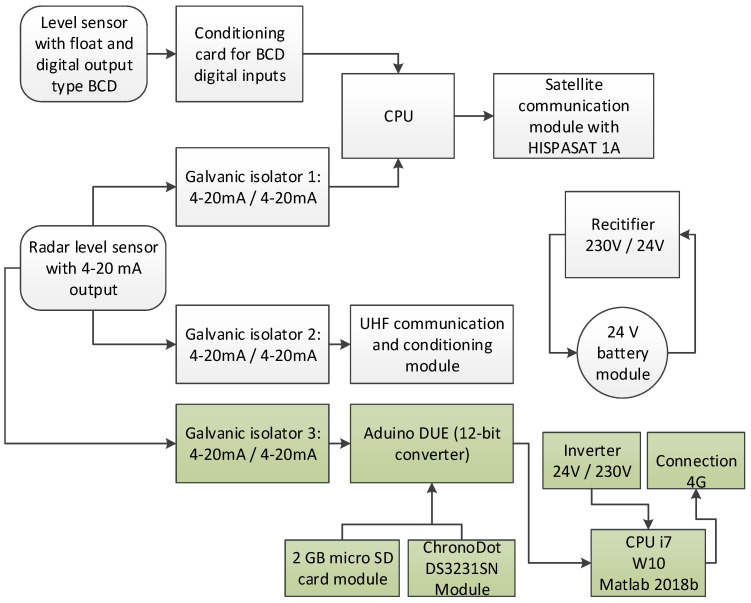
Block diagram of the SAIH system (SAINCO/Telvent) and the equipment developed (green).

An electronic equipment has been developed with capabilities similar to the SAIH equipment for signal capture, conditioning, storage and sending of the same data in remote connection. In the block diagram of Figure 7, the original set of equipment the one proposed here are illustrated.

**Figure 8:**
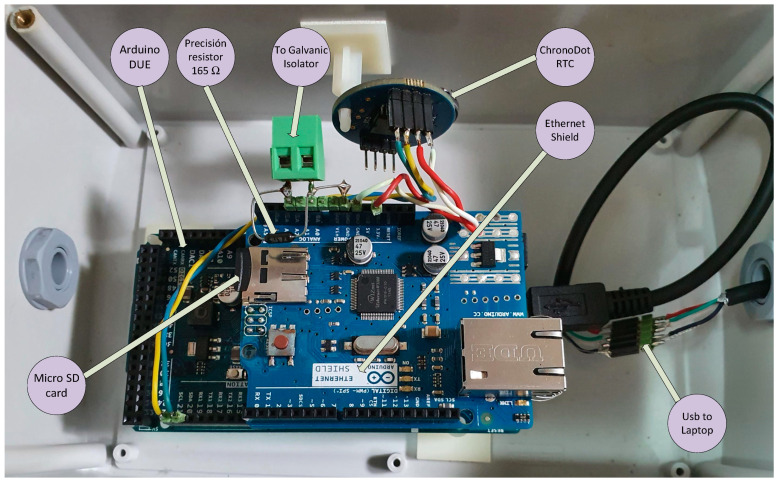
Equipment implemented for the test at the A17 Genil-Écija control point of the SAIH system of the Guadalquivir river.

The proposed system has the advantage that, in addition to the functions described above, it can fill in incomplete data series and validate them in real time. The elements of this system (Figure 8) are:

1. ABB CONTROL model 1SVR011718R2500 galvanic isolator [59] powered at 24 V DC, with input and outputs in the 4–20 mA range
2. Arduino DUE module with a 32-bit Atmel SAM3 × 8E ARM Cortex-M3 CPU microcontroller [60], with a 12-bit analog/digital converter (A/D) and 0–3.3 V measurement range
3. Arduino DUE also has a USB connection for a virtual RS232C port through which it obtains power
4. This microcontroller has several input/output ports, it will be connected to the memory module (micro SD) with the serial peripheral interface (SPI) protocol and with the clock-calendar module with the inter-integrated circuit (I^2^C) protocol. In both cases, the signal voltage will be 3.3 V
5. Precision resistor of 165 Ω, 0.25 W, ± 0.1% precision and ± 15 ppm/° C, [61], to go from 4–20 mA current levels to voltages between 0.66 V and 3.3 V, (V = IR)
6. Ethernet module with a micro SD card socket [62], compatible with 3.3 V level signals, and with a W5100 Ethernet controller for local area network (LAN) communications. For the configuration that has been used, it only requires an SPI connection to access the micro SD card, which is used to record the data including the date and time they were acquired
7. ChronoDot Real Time Clock (RTC) module [63], which is a temperature compensated calendar clock based on the DS3231SN chip with a drift of only ± 2 pmm, (1 min per year). It includes a CR1632 lithium battery, which gives it autonomy for about 8 years, being compatible with I^2^C signals of level 3.3 V
8. Single-phase inverter from 24 V DC to 230 V AC of 300 W model A301-300W-24 [64] with square wave output at 50 Hz, ideal for supplying current to the power supply of a laptop
9. Huawei 4G USB Modem model ES3372 [65] for internet connection
10. Laptop with i7 processor, 8GB of RAM, Windows 10 and Matlab 2018b

**Figure 9:**
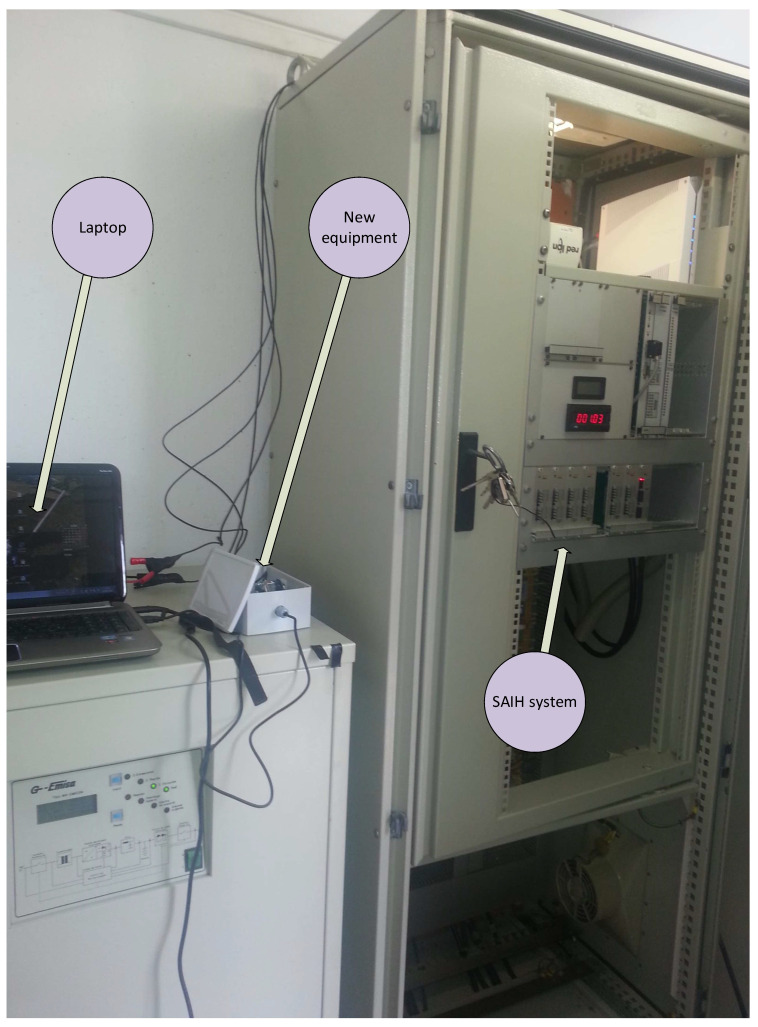
The new equipment coupled to the SAIH system.

The signal from the sensor through the 4–20 mA current loop is copied by the galvanic isolator, transformed into the input of the A/D converter to voltage through a 165 Ω resistor and finally over-sampled (1000 times in each acquisition) to obtain its average value, so that the system acts as a low-pass filter that eliminates possible electrical noise from the signal. The acquisition has a cadence of one second. Each new data is stored on the micro SD card together with the date and time from the ChronoDot module. Subsequently the data is sent to the laptop through the virtual serial port generated with the USB connection. This equipment is autonomous and repeats this process continuously, regardless of whether the computer processes the information or not, thereby ensuring that the information acquired remains intact and ready to be read at any time from the memory card. Figure 9 shows the system together with the SAIH equipment.

#### 2.6.1. Calibration of the Developed Equipment

To calibrate the developed equipment, a HT8000 digital process calibrator [66] has been used, which applies known intensity values in the 4–20 mA range to the current loop. With the calibration data obtained, the adjustment data provided by the SAIH for point A17 Genil-Écija, 4 mA for the 0 m level and 20 mA for the 10.71 m level, have been applied. Calibration results appear in Appendix 5 [56].

#### 2.6.2. Implementation in LCPs

As it has discussed above, the use of small processors, are justified not only due to their low cost but also due to their technical advantages in the management of this kind of data. These data need a smaller storage size, a small battery, with a lower maintenance cost, all of which are an advantage from the economical point of view and collection of quality data set. The developed software is in Appendix 6 [56].

#### 2.6.3. Using Arduino

Two models of Arduino has been used in this work, Arduino UNO (FLASH = 32kB, SRAM = 2 kB, CLK = 16 MHz), and Arduino DUE (FLASH = 512 MB, SRAM = 96 kB, CLK = 84 MHz), in order to compare their capacity with these algorithms and their electrical consumption.

The data series to be validated come from a laptop connected to Arduino. These data are sent to the Arduino basic board in real-time to be validated. After the validation process, the data series is received by the laptop. This process depends on the processing speed of the evaluated board.

Firstly, the implementation of the Splines in Arduino has been developed following a sketch from [67]. This is a simple library for different types of 1-D Splines, written for the Arduino environment. Secondly, the implementation of RBF in Arduino was developed following [68] and [69]. Finally, for MLP implementation the developed sketch is based on [70]. The method chosen to fit weight coefficients in this neuronal network has been the simulated annealing algorithm [71].

#### 2.6.4. Use of Raspberry Pi 3

All the functions related to Splines, RBF and MLP are developed using the same source in Arduino, but in this case with an adaption to Python 2.

### 2.7. Previous Simulation in Matlab

The techniques further explained ahead have been ran previously on MatLab. With the aim of measuring their capabilities in real time process, these techniques were written under Arduino programming language (a specific language for Arduino, it is a high-level processing language similar to C++). Regarding Raspberry Pi 3, the language used has been Python, which comes from the free operating system based on Debian called Raspbian. The main characteristics of the three basic electronic boards used in this work are summarized in the Table 3.

### 2.8. Methods Used for IoT Connection

For the distribution of the data over the internet (using 4G), a shared folder in DropBox is used [72]. On the other hand, the remote control of the laptop located at the control point is done through the LogMeIn Pro application [73].

The data were validated with a 24-h cadence, using either remote access to the latptop or the reading of the file stored in DropBox.

To analyze the different gap-filling methods proposed and their accuracy, two approximations were considered. The first one, Test I, (Section 3.1) is designed for scattered gaps, and the second one, Test II, (Section 3.2) for multiple gaps. In Section 3.3 Test III has been carried out for comparison of the three different boards. In the same section Test IV has been performed to check the processing speed on RPi3 with MLP = (50 50 5). In Section 3.4 the data from the control point A17 Genil-Écija are analyzed. In Section 3.5 the quantification of the maximum resolution of the dynamic NARNN is studied. Finally in Section 3.6 the computational cost of real-time pre-validations is analyzed.

## 3. Results

### 3.1. Test I

The goodness of fit found was with the application of the three methods suggests a ranking such as Splines > RBF > MLP. The SEE values obtained for the spline technique and RBF methods are one order of magnitude lower than for MLP method (Table 4). The scant efficiency of the MLP is attributed to the monolayer perceptron, with a small number of neurons in their hidden layer, *n* < 10. The latter structure in the MLP tends to become generalized by establishing behavior patterns, while the other two methods only take into consideration the value of the known data to make the gap-filling estimation. Therefore, the MLP method performs worse than the other methods.

On the other hand, in an MLP with a larger number of neurons, the differences between the three methods decrease. Thus, a mono layer perceptron (ANN), with *n* = 50, gives similar results to splines and RBF functions. In fact, if the number of hidden layers is raised to 2 or 3, the results become equal and, in some cases, the results are better in the perceptron than in the other two methods. In this case, the MLP has lost its capacity to generalize in favor of learning by a route that is good for the estimation in small gaps. Figure 1, Figure 2 and Figure 3 in Appendix 7 [56] depict the growing complexity of ANN in its adaptive behavior.

**Figure 10:**
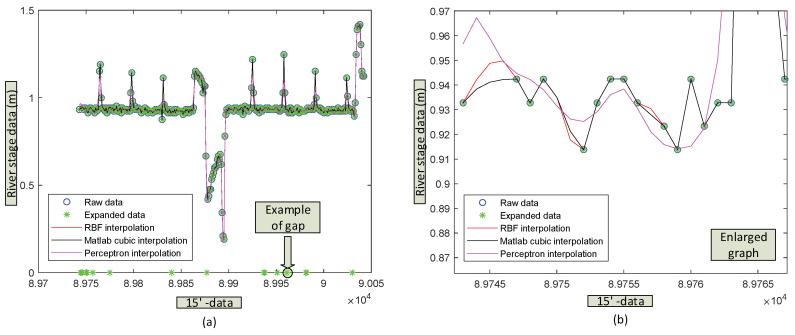
(**a**) Comparison of the three gap-filling methods: RBFs, Cubic spline and mono layer perceptron (50 50 5), (80 10 10). (**b**) Details of interpolations.

The behavior of the three methods for the case of a perceptron with a more complex structure, (MLP 50-50-5: 50, 50 and 5 neurons on the first, second and third layer, respectively) (Figure 10), is shown in detail in Table 4 for the values of the parameters *n* = 300, *m* = 0.10 and *p* = 0.80. The ANN has been trained with segmented data (80 10 10) fitting the weighting coefficients to the Levenberg-Marquardt (LM) algorithm.

At this level of complexity in the ANN structure, the SEE found in the three methods shows the same order of magnitude, but at a greater computational cost for the MLP. This fact represents a drawback for practical use for real time computing.

The spline technique is more versatile than the other methods for the estimation in scattered gaps. MLP has to reduce its capacity for generalising, but the increase in the memory capacity of the MLP demands a high computing cost, which impairs its practical use. RBF methods perform similarly to splines in all cases, but at a higher computing effort, which excludes it as an gap-filling method.

### 3.2. Test II

The foregoing analysis indicated that, in the case of multiple gaps, MLP could be as good, or even better than splines as interpolating tools. The option analyzed in Test I requires a generalized coupling of the ANN with a minimum of memory. To confirm this conjecture, a further test with only one neuron in the hidden layer was made. For this test, a sample of 100 iterations, with the random example size ranging between 100 and 5000 registers from the dataset, *m* = 0.05 as fraction of gaps, and *n*_e_ = 1 neuron were taken.

The results obtained are shown in Table 4, in which the rows are the fractions of gaps used, p (0.05 0.75), and the columns the sample size used *n* (100 5000). The ratio between the respective errors from the splines and the perceptron methods (SEE cubic splines/SEE MLP) appears in the cells. These ratios confirm the initial speculation regarding its gap-filling capacity: perceptron exceeds splines in multiple gaps. As summarized in Table 5, there is a proper zone for each of the techniques. The not bold area stands for the spline while the bold area represents the perceptron results.

As can be observed in this table, when the sample size is small (*n* = 100), the perceptron behavior is better than the spline method with multiple gaps (75%). On the contrary, when the sample size increases, MLP performs better and it is able to fill gaps increasing its accuracy. For sample size *n* > 5000, the gap-filling capacity of MLP is greater than that of the splines, irrespective of the gap size (*p*).

### 3.3. Results of the Use of Boards Based on Arduino and Raspberry Pi 3

The use of Arduino UNO has quite limitations because data processing needs to use RAM memory, and this one is quite limited (2 KBYTE). Moreover, the use of floating point is limited as well as the maximum speed processing (16 MIPS). In the case of Arduino DUE, the RAM memory is up to 96 KB. Programs are executed with 32 bits and 84 MIPS. The main limitation in both cases is the sample size data, which is conditioned on the available RAM memory.

#### 3.3.1. Test III

In order to carry out an effective comparison of the three different boards (UNO, DUE and RPI3), a 30-data sample size has been selected. This sample size has a gap fraction *m* = 0.1, so only a maximum of three data could be missing. In the case of finding *m*1 = 2 missed data, being the fraction to be marked *p* = 1, then the number of data to be marked with the flag 6 would be q = 2 × 1 = 2, which are used to measure the goodness-of-fit in each case.

This dataset is sent to the boards from the laptop running a MatLab application, which receives back the results of this process to record in a text file to be studied later.

The results from the two first processes (Splines and RBF) are similar to the results obtained from MatLab, except for the runtime needed. The implementation of a large ANN is very difficult in this kind of low-cost architecture, Arduino UNO and DUE, due to the lack of memory and computer power within a reasonable time.

As comparative test, a perceptron (only one layer and 5 neurons) has been set up for these three boards. The simulated annealing process, in the case of Arduino, uses a random number generator (RNG), whose initialization is utterly random. Table 6 shows the measured time directly from MatLab in each case under study.

#### 3.3.2. Test IV

This test has been carried out only with Raspberry Pi3, under equal conditions as in MatLab, in order to check the processing speed. 

A MLP = (50, 50, 5) has been tested, taken several sample size with different *p* and *n*, every one of them with a 15-min data. Table 7 shows the results obtained in this test.

### 3.4. Alternative Pre-Validation System with IoT: Analysis of the Data of the Tests Carried Out in the Control Point A17 Genil-Écija

The tests carried out began on 15 April 2019 with the installation of the equipment, tests of the 4G connection, analysis of the integrity of the signal from the sensor through the galvanic separator and configuration of the remote desktop.

On 24 April 2019, tests were carried out on the 24 V supply, with which it was estimated whether the inverter could influence the quality of the signal from the sensor, and whether a direct supply from the 230 V grid could be of interest. From the results it was confirmed that the inverter did not alter the quality of the signal, a predictable result given that the sensor sends its signal in a current loop.

On 1 May 2019 at 00:00:00, the data collection of the radar level sensor began; This data collection was recorded in the micro-SD memory and downloaded through the RS232C port on the hard disk of the laptop and simultaneously through the 4G connection in a shared DropBox folder. During this process, the correct operation of the equipment was controlled by remote desktop, proceeding to the daily validation by an expert of the data obtained. On 21 May 2019 at 1:51:13 p.m., data collection ceased, the equipment was removed and the trial was terminated. During this time interval, no error was detected in the data validation of the 1,777,874 data recorded at a rate of one second.

**Figure 11:**
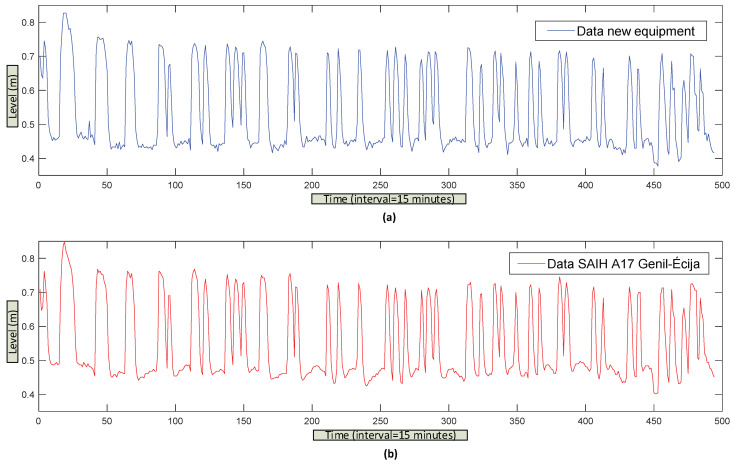
Data obtained (every 15 mins from the A17 Genil-Écija control point), (**a**) with the equipment developed and (**b**) with those from the SAIH of the Guadalquivir river.

Figure 11a,b show the data corresponding to these 3 weeks of trials. In Figure 11a, the data obtained by the installed equipment and in Figure 11b, those from the SAIH. Given that the data from the SAIH has a cadence of 15 min, the data from the development equipment have been filtered in Figure 11a so that its cadence is the same, synchronizing them approximately with those of the SAIH (xx:00 h, xx:15 h, xx:30 h and xx:45 h).

It is noteworthy that the remote station SAP20 (SAINCO/Telvent equipment) sends the data with a resolution of 12 bits and a cadence of approximately one minute. The SCADA of the basin processing center (CPC) adds the approximate time to the data, so Figure 11a,b are not exactly the same.

### 3.5. Alternative Pre-Validation System with IoT: Quantification of the Maximum Resolution of the Dynamic NARNN Based on its Configuration Parameters

**Figure 12:**
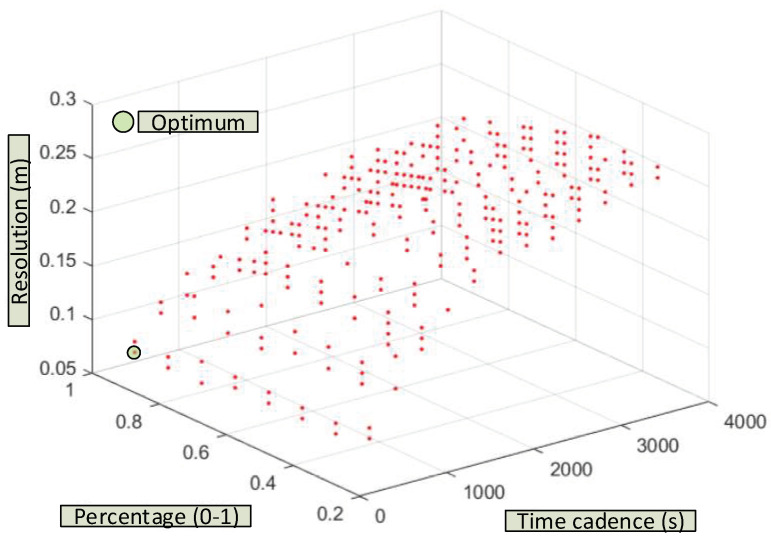
Resolution of the dynamic NARNN as a function of the selected time interval and the percentage of data used for validation.

**Figure 13:**
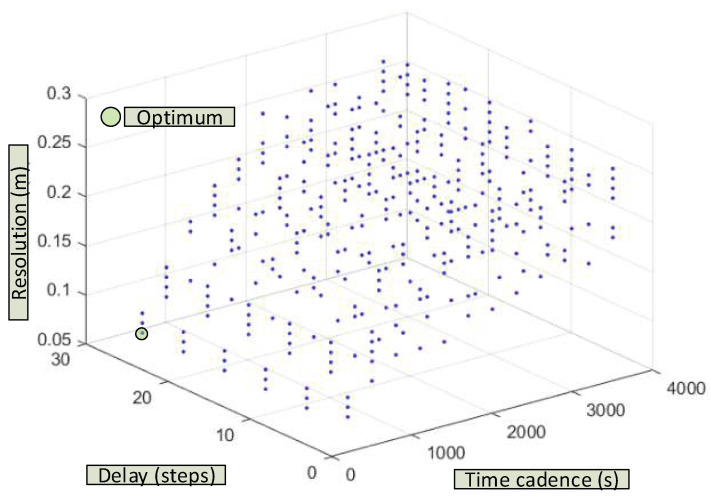
Resolution of the dynamic NARNN as a function of the selected cadence and the lag (or delay) used in the NARNN feedback.

Multiple dynamic NARNN performance simulations have been carried out with different parameters: cadence between data (3600 s,..., 60 s), the feedback delay (1, 6, 11, 16, 21 and 24), and finally the ratio between the data used for training and the total of those acquired (0.25,…, 0.95). The difference up to 1 (0.75,…, 0.05) is the percentage that would be used for validation. The results appear in Figure 12 and Figure 13. Complementary information can be found in Appendix 8 [56].

As can be seen in Figure 12, the best results are obtained for short cadences (5 min), and percentages of data destined for training of 95%. In these circumstances, the resolution of the dynamic NARNN is 8 cm. In the opposite case with hourly cadences and 25% of data intended for training, the resolution of the dynamic NARNN is reduced to 26 cm.

In Figure 13, the results for different cadences and feedbacks of the NARNN inputs are shown. The best results (9 cm) are obtained for 5 min cadences and high delays (26). The worst results again correspond to hourly cadences and in this case with unit delay (27 cm). In the most optimal case, (cadence 300 s, delay 26 and percentage 95%), a resolution of 7 cm is reached. Simulations representing a large computational effort have been carried out for cadences of 60 s. Table 8 shows some of the results obtained.

As can be seen, the greater the delay and the greater the percentage of data used for NARNN training, the better the resolution values obtained. Thus, for example, for a delay 30 and percentage of data for validation 95%, a resolution of 5.5 cm is reached.

### 3.6. Alternative Pre-Validation System with IoT. Computational Cost of Real-Time Pre-Validations

The duration of the pre-validation process in real time for the different configurations of the NARNN has been evaluated, considering, as already mentioned, that the operating system of a computer is not real time (RTOS), therefore it has been established a margin of safety in such a way that:

(11) $$t_{\mathrm{cadence}}\geq 2\cdot t_{\mathrm{processing}}$$

Table 9 shows the results obtained with this restriction.

A laptop with an Intel ^®^ Core ™ i7-2670CQ @ 2.20 GHz processor, with 8GB of RAM and 64-bit and Windows 10 was used. As described above (Table 9), case (1) to (2) or (4), and case (3) to (5) will be preferable. Therefore, an attempt will be made to select as optimal (from a calculation effort point of view) between cases (1), (3) and (6). The one that provides a lower value (resolution) is preferable. Table 10 shows the results obtained in the simulation.

If it does not interest that the cadence is a whole fraction of the normal times of the SAIH (15 min and one hour), the case (3) that offers the best resolution is preferable. Otherwise, case (6) would be preferred, since being an interval of whole minutes, it is always an integral fraction of any normalized interval.

## 4. Conclusions

In this work, as first contribution, a new assessment of different techniques for restoring missing river stage data is proposed. Due to the increase of extreme events occurrence and, in order to improve the management of water resources, complete river-stage time series are needed. This process has gained great importance for scientific or technical applications, and especially -in the current climate change context of hydrologic models running in Decision Support Systems. In addition, the development of specific methods allowing one to complete the gaps in hydrologic datasets appropriately will improve their reliability and increase the quality of the results from different climate or hydrologic works that generally use these data as inputs.

To restore the full river stage data series three gap-filling methods have been studied, showing that it is sufficient to use cubic splines for scattered gaps and monolayer perceptrons with a small number of neurons for multiple gaps.

The use of ANNs is not recommendable for scattered gaps due to its tendency to generalize and its high computing cost. The use of RBFs, more complex than splines, does not appreciably improve the latter’s efficiency. Therefore, RBF is not advisable for its use in gap-filling river stage data.

The best methods according to the assessment carried out in this work are: splines and mono-layer perceptrons. Regarding their ability to run in low capacity processors with low electrical consumption, both gap-filling methods can be realized on low-cost architecture devices (e.g., Arduino and Raspberry Pi), allowing easy positioning at both single and multiple locations, once the software has been optimized.

In this case and without any optimization of the software, it has been verified that this kind of architecture based on Arduino, especially UNO, is not suitable for perceptron. Regarding to Raspberry Pi3, its use could be limited in this kind of test with large sample size or large gaps in the data series.

The methods proposed here can be applied to the handling of other hydro-meteorological variables, such as temperature, relative humidity or precipitation. The optimal method, in each case, would depend on the nature and quality of the data set, sensor characteristics as well as the collecting data process used. Future works will be focused on the application of these techniques to various control points simultaneously along the river axis in order to study individual cases like the existence of flood control reservoirs.

As a second contribution, an IoT equipment has been developed, which has been installed in a SAIH control point, to evaluate the possibility of incorporating a river level data pre-validation system, based on a non-linear neural network auto- Regressive (NARNN), with a dynamic training seed, tests have shown that it works well.

The behavior of this NARNN, in terms of its ability to discern in real time between valid and erroneous data, improves with a lower cadence between data, greater feedback and a greater number of training data.

The duration of the process in each configuration allows proposing two alternatives depending on the compatibility sought with the standardized data obtained by the SAIH.

These results allow us to affirm that it is possible to develop a processing equipment, with a set of management programs, that is capable of independently validating, (i) for cases in which the sensor has stopped working for a while, using the methods of data filling shown in this work and (ii) pre-validating in real time using a dynamic seed NARNN.

## Figures and Tables

**Table 1:** Consumption and time-comparison of different low capacity processors (LCPs).

Consumption (Watts)				Duration (Number of Times)				
PC	Raspberry Pi (RPi)	Arduino DUE	Arduino UNO	RPi/PC	Arduino DUE/PC	Arduino UNO/PC	Arduino DUE/Rpi	Arduino UNO/Rpi
220	1.8	0.8	0.4	122	220	550	2	4.5

**Table 2:** Different flags in data records.

Flag	Type of Data
0	Correct
1	None
2	No satellite connection
3	Out of range
4	Manual
5	Non-observed-change in time interval

**Table 3:** Main characteristics of the three basic electronic boards used in this study.

Board	Processor	Bits	MIPS	SO
Arduino UNO	ATMEGA328P-PU	8	16	NO
Arduino DUE	SAM3 × 8E ARM Cortex-M3	32	84	NO
Raspberry PI 3	Broadcom BCM2837	32	2441	RASPBIAN

**Table 4:** Comparison between spline, RBF’s and perceptron (50 50 5), (80 10 10), (LM) with (*n* = 300, *m* = 10%, *p* = 80%).

						SEE						
*n*	*m*	*p*	*m*1	q	Neurons	Spline (×10^−2^)	RBF_Lin (×10^−2^)	RBF_G (×10^−2^)	RBF_C (×10^−2^)	RBF_T (×10^−2^)	RBF_M (×10^−2^)	MLP (×10^−2^)
300	10	80	10	8	50_50_5	1.32	1.46	3.96	1.76	1.66	1.46	3.58
300	10	80	30	24	50_50_5	10.5	5.01	8.30	8.90	7.63	5.01	7.75
300	10	80	13	10	50_50_5	2.01	2.78	6.69	4.33	3.60	2.78	1.84

**Table 5:** Comparison between cubic splines and MLP in multiple-gap interpolation (cubic spline-MLP SEE ratio).

p/n	100	150	200	500	1000	5000
0.05	0.623	0.642	0.771	0.89	0.972	**1.03**
0.10	0.71	0.872	0.948	0.99	**1.09**	**1.06**
0.25	0.914	0.955	**1.11**	**1.14**	**1.11**	**1.11**
0.50	0.99	0.998	**1.08**	**1.13**	**1.06**	**1.13**
0.75	**1.06**	**1.06**	**1.07**	**1.09**	**1.15**	**1.04**
* Bold font for MLP						

**Table 6:** Processing time in Arduino UNO, DUE and Rapsberry Pi3.

Board	Spline	RBF Lineal	RBF Gaussian	RBF Cubic	RBF Thin-Plate	RBF Muticuadrics	MLP (*n* = 5)
Arduino UNO	<2 s	<1 s	<3 s	<1s	<1s	<3s	<45 min
Arduino DUE	<1 s	<1s	<1s	<1s	<1s	<1s	<8 min
Raspberry PI 3	<1 s	<1s	<1s	<1s	<1s	<1s	<1min

**Table 7:** Processing time in Test IV with RPi3.

p/n	100	150	200
0.05	<8 min	<14 min	<21 min
0.1	<12 min	<18 min	<32 min
0.25	<17 min	<24 min	< 51 min

**Table 8:** Simulations for 60 s cadences.

Cadence (s)	n_Neurons	Delay	Percentage (%)	Resolution (cm)
60	1	1	25	9
60	1	1	35	9
60	1	11	25	9
60	1	21	95	7.5
60	1	26	25	8
60	1	30	95	5.5

**Table 9:** Duration of the pre-validation processes with 1 neuron and delay 1.

Case	Cadence (s)	Percentage (%)	Time Cost (s)
1	50	0.25	23.1
2	55	0.25	24.5
3	55	0.35	26.1
4	60	0.25	20.0
5	60	0.35	28.3
6	60	0.45	27.5

**Table 10:** Resolution in optimal cases with one neuron and delay 1.

Case	Cadence (s)	Percentage (%)	Time Cost (s)	Resolution (cm)
1	50	0.25	23.1	8.53
3	55	0.35	26.1	6.14
6	60	0.45	27.5	8.32
